# Critical Assessment of a Structure-Based Screening Campaign for IDO1 Inhibitors: Tips and Pitfalls

**DOI:** 10.3390/ijms23073981

**Published:** 2022-04-02

**Authors:** Andrea Mammoli, Elisa Bianconi, Luana Ruta, Alessandra Riccio, Carlo Bigiotti, Maria Souma, Andrea Carotti, Sofia Rossini, Chiara Suvieri, Maria Teresa Pallotta, Ursula Grohmann, Emidio Camaioni, Antonio Macchiarulo

**Affiliations:** 1 Department of Pharmaceutical Sciences, University of Perugia, Via del Liceo n.1, 06123 Perugia, Italy; andrea.mammoli@chimfarm.unipg.it (A.M.); elisa.bianconi@chimfarm.unipg.it (E.B.); luana.ruta@studenti.unipg.it (L.R.); alessandra.riccio1@studenti.unipg.it (A.R.); carlo.bigiotti@studenti.unipg.it (C.B.); maria.souma@studenti.unipg.it (M.S.); andrea.carotti@unipg.it (A.C.); emidio.camaioni@unipg.it (E.C.); 2 Department of Medicine and Surgery, University of Perugia, P.le Gambuli, 06132 Perugia, Italy; sofia.rossini@studenti.unipg.it (S.R.); chiara.suvieri@studenti.unipg.it (C.S.); maria.pallotta@unipg.it (M.T.P.); ursula.grohmann@unipg.it (U.G.)

**Keywords:** fragment-based drug design, molecular docking, biophysics, virtual screening, thermophoresis

## Abstract

Over the last two decades, indoleamine 2,3-dioxygenase 1 (IDO1) has attracted wide interest as a key player in immune regulation, fostering the design and development of small molecule inhibitors to restore immune response in tumor immunity. In this framework, biochemical, structural, and pharmacological studies have unveiled peculiar structural plasticity of IDO1, with different conformations and functional states that are coupled to fine regulation of its catalytic activity and non-enzymic functions. The large plasticity of IDO1 may affect its ligand recognition process, generating bias in structure-based drug design campaigns. In this work, we report a screening campaign of a fragment library of compounds, grounding on the use of three distinct conformations of IDO1 that recapitulate its structural plasticity to some extent. Results are instrumental to discuss tips and pitfalls that, due to the large plasticity of the enzyme, may influence the identification of novel and differentiated chemical scaffolds of IDO1 ligands in structure-based screening campaigns.

## 1. Introduction

**Figure 1:**
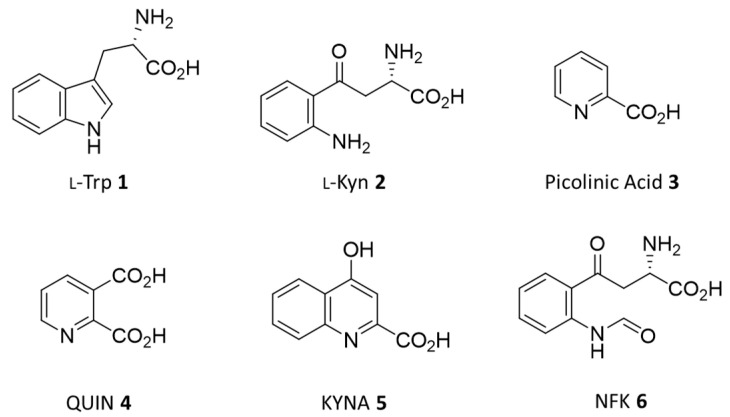
Substrate (**1**) and metabolites (**2**–**6**) of the kynurenine pathway.

l-Tryptophan (l-Trp, **1**; Figure 1) is an essential aromatic amino acid participating in synthesis of proteins and bioactive metabolites in mammals [1]. Specifically, the majority of l-Trp from the diet is catabolized in the kynurenine pathway (KP) for the generation of immunoactive metabolites (KP) [2], such as l-kynurenine (l-Kyn, **2**) and picolinic acid (PIC, **3**), and neuroactive metabolites, such as quinolinic acid (QUIN, **4**) and kynurenic acid (KYNA, **5**) [3].

Two major enzymes, tryptophan 2,3-dioxygenase 2 (TDO2) and indoleamine 2,3-dioxygenase 1 (IDO1), catalyze the initial and rate-limiting step of KP, promoting the oxidative cleavage of the pyrrole ring of the indole nucleus of l-Trp (**1**) to yield N-formyl-kynurenine (NFK, **6**) [4,5,6]. These proteins display different specificity and affinity to l-Trp (**1**), with IDO1 showing a wider substrate promiscuity to indole-bearing compounds, but a lower affinity to l-Trp (**1**) than TDO2 [7,8,9]. Moreover, they also have a characteristic tissue distribution and enzyme-specific pattern of post-translational modifications. In particular, TDO2 is mainly present in the liver and cholangiocytes, regulating l-Trp homeostasis; IDO1 is ubiquitously expressed in many tissues and its levels are found to be augmented in multiple cancers being correlated to poor prognosis [10,11]. Concerning enzyme-specific post-translational modifications, IDO1 bears two functional immunoreceptor tyrosine-based inhibition motifs (ITIM, Tyr111, Tyr249) and a YENM motif (residues 145–148) that are associated with the regulation of the non-catalytic signaling functions and subcellular localization of the enzyme, respectively [12,13].

**Figure 2:**
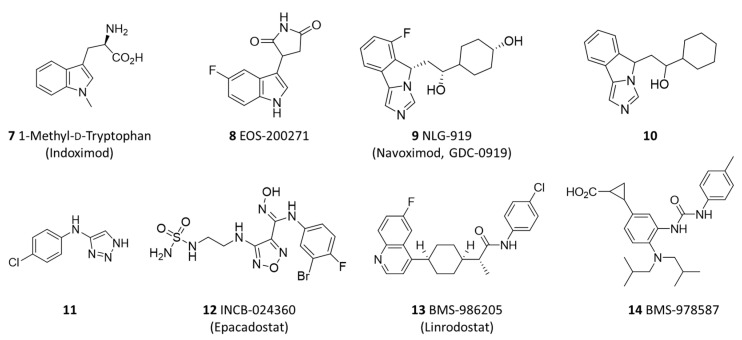
Chemical structures of selected indoleamine 2,3-dioxygenase 1(IDO1) inhibitors (**7**–**14**).

Over the past two decades, researchers have paid close attention to IDO1 as a drug target involved in the molecular mechanisms of tumor immune escape [14,15,16]. This has fostered the design of catalytic inhibitors of IDO1 (**7**–**14**; Figure 2) for the development of immunotherapeutic drugs that make tumor cells more vulnerable to T cell detection and destruction [17,18]. Despite the large amount of research devoted to the design and synthesis of such compounds [19], few IDO1 inhibitors have entered clinical trials (**7**–**9**, **12**, **13**) and recent disappointing results bolster the quest for novel IDO1 inhibitors with more efficacious pharmacological and clinical profiles [20,21,22].

**Figure 3:**
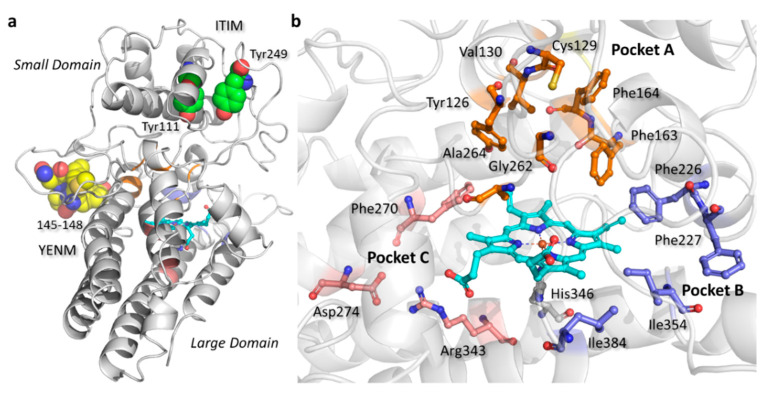
(**a**) Structure and post-translational modification sites of IDO1; (**b**) ligand binding pockets of IDO1. Images have been generated with the software Maestro, using the PDB entry 6E45 after its processing with the Protein Preparation Wizard (PPW) tool.

In this framework, the availability of numerous IDO1 crystallographic studies has prompted the structure-based design as a method of choice to identify and optimize inhibitors bearing different chemical scaffolds and mechanisms of binding to the enzyme [23,24,25,26,27,28,29,30,31,32,33,34,35,36,37,38]. These works have unveiled a conserved fold of the IDO1 primary sequence, which is composed of a large catalytic domain and a small non-catalytic domain (Figure 3a). The large domain holds the heme group and contains the catalytic cleft, which is composed of pockets A and B that are placed over the sixth coordination site of the porphyrin iron and nearby the entry to the heme binding site, respectively. In particular, pocket A (Figure 3b) is defined by hydrophobic and very few polar residues (Tyr126, Cys129, Val130, Phe163, Phe164, Gly262, and Ala264) [24], whereas pocket B (Figure 3b) is shaped by hydrophobic residues (Phe226, Phe227, Ile354 and Leu384) [25]. A further allosteric site, named pocket C (Figure 3b), is located below the heme plane and accounts for the binding of positive allosteric modulators and the inhibition by substrate phenomenon [38,39]. This additional pocket is defined by few hydrophobic and charged polar residues (Phe270, Asp274, Arg343).

**Figure 4:**
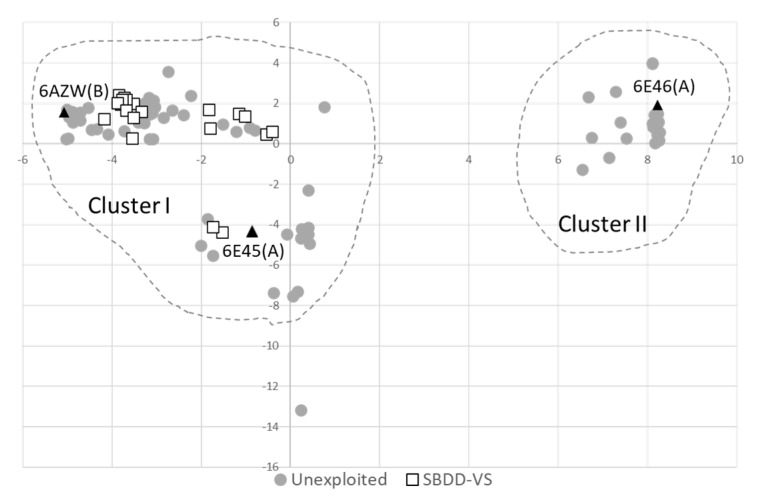
Molecular recognition space of IDO1. The two clusters of conformations of the catalytic cleft are highlighted with dashed lines (Cluster **I** and **II**). PDB entries (and chain) of IDO1 structures used in this study are indicated with black triangles and labelled. PDB entries that have already been employed in structure-based screening campaigns are indicated with white boxes; other unexploited entries are indicated with grey circles. Data were taken from reference [40].

Recently, we have performed an analysis on the structural motifs and molecular recognition properties of the available ligand-bound and unbound structures of IDO1 [40]. As a result, we identified two clusters of conformations of the catalytic cleft (pockets A and B) which are endowed with different steric shapes and hydrophilic features. These conformations occupy distinct regions at the edges of the molecular recognition space of IDO1 (Figure 4).

As a follow-up to our efforts in investigating the molecular aspects of ligand recognition by IDO1, herein we report the critical assessment of a structure-based screening campaign that integrates molecular docking of a library of fragment molecules with biophysics and cellular assays. For this study, three distinct conformations of the catalytic cleft were used as input structures for molecular docking in order to assess whether and to what extent their diverse steric and hydrophobic features affect the diversity and number of the hit compounds identified.

## 2. Results

### 2.1. Selection of IDO1 Structures

In accordance with the results of our previous study [40], three IDO1 chain structures were selected from different regions of the molecular recognition space of the enzyme (Figure 4). Criteria of selection were: (i) coverage of a specific and diverse region of IDO1 molecular recognition space; (ii) no employment in previous structure-based screening campaigns; (iii) best resolution factor compared to neighboring IDO1 structures.

As a result, chain B of 6AZW (apo-form, inhibitor bound and heme-free structure) [35] (6AZW), chain A of 6E45 (holo-form, heme-bound structure) ([36], 6E45) and chain A of 6E46 (holo-form, substrate-bound structure) ([36], 6E46) were selected for the virtual screening campaign.

Binding site properties of the catalytic cleft in the three IDO1 chain structures show that they differ in size and hydrophobic balance, with 6AZW being larger and more hydrophobic than 6E45 and 6E46, as well as in the hydrogen-bond interaction expected from a bound ligand, with 6E45 preferring ligands making hydrogen-bond donor interactions, and 6E46 expecting compounds with hydrogen-bond acceptor interactions (Table 1; Figures S1–S3, Supplementary materials).

Since these different properties of the binding site may affect the outcome of a structure-based screening campaign, the selected IDO1 structures were used as input to perform three parallel virtual screening studies and evaluate the extent to which these properties affect the number and diversity of resulting hit compounds.

### 2.2. Structure-Based Virtual Screening

A chemical library of 44,000 compounds was selected from the Life Chemicals collection, applying fragment-like criteria (*M*_W_ ≤ 300 and _calc_logP ≤ 3.0) [41,42,43]. The chemical structure of each of these compounds was docked into the catalytic cleft of the three IDO1 structures, using Gscore (kcal/mol) to assess its binding energy (see methods for further details). Compounds showing a Gscore better than −4 kcal/mol were selected from each docking run and clustered applying the K-means method based on radial Morgan extended-connectivity fingerprints (ECFPs) [44]. Specifically, the use of such a cutoff value allowed the selection of 99.4% (6AZW), 76.3% (6E45), and 68.3% (6E46) of top scored compounds amid successfully docked ligands in each IDO1 structure (Figure S4, Supplementary materials).

A total of 23 clusters was generated for each set of selected compounds (set A/6AZW, B/6E45, and C/6E46), yielding a total of 69 clusters. One representative compound for each cluster was then identified and purchased, according to the criteria of better binding energy (Gscore, kcal/mol), visual inspection, and commercial availability.

Analyses of the average of polar surface areas (PSA) and statistical mode of the number of hydrogen bond donors and acceptors of the three sets of representative compounds (Table 2) show a complementary compound with the different properties of the IDO1 binding site which was used for the relative docking study. Specifically, compounds with lower PSA are recovered in top scored solutions when docked against the more hydrophobic cleft of the 6AZW structure. Interestingly, this set of compounds shows a better average value of binding energy scores over the other two sets of compounds. In contrast, virtual hits identified by docking into the 6E45 structure are featured on the statistical mode by a higher number of hydrogen bond donor and acceptor groups which, however, do not yield an improvement in ligand binding energy scores.

**Figure 5:**
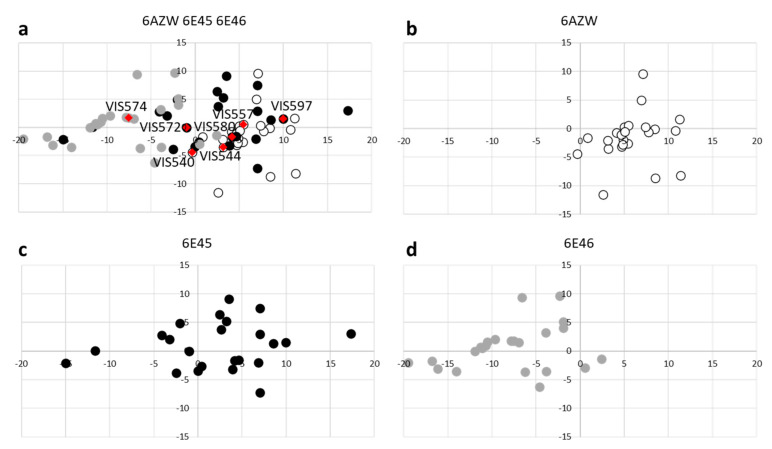
Score plot of the PCA study that was carried out using topological descriptors calculated on the entire set of 69 virtual hits (**a**); confirmed IDO1 ligands are labelled and highlighted as red diamonds. PCA scores of compounds belonging to set A, B and C are then extracted and plotted with the additional panels (**b**–**d**), respectively. Graphs are generated plotting the first component (PC1) and second component (PC2) values on x and y axis, respectively.

The diversity covered in the ’chemical space’ by each set of compounds can be easily visualized with the score plots resulting from a principal component analysis (PCA). Specifically, one PCA study was carried out on the entire set of 69 compounds, calculating 199 topological descriptors for each molecule. The first two principal components (PC1, PC2; Figure 5) explain the 91% of the variance of the original set of descriptors.

The inspection of the score plot shows that compounds belonging to set A mostly occupy positive values of PC1, compounds of set B spread from positive to negative values of PC1, while compounds of set C lie in the region of negative values of PC1. Hence, in the framework of a topological description of the chemical structure, compounds of set A (virtual screening on chain B 6AZW, inhibitor bound and heme-free structure) show remarkable chemical diversity from compounds of set C (virtual screening on chain A of 6E46, substrate-bound structure). Conversely, compounds of set B (virtual screening on chain A of 6E45, heme-bound structure) include chemotypes spanning a wider area of diversity that comprises both regions covered by molecules of set A and set C in the chemical space. These observations support the idea that diverse steric and hydrophobic features of the three distinct conformations of IDO1 catalytic cleft affect the chemical diversity of the identified virtual hit compounds.

### 2.3. Experimental Binding Studies

The next step of the study was to confirm the number of true binders among the 69 virtual hits. Therefore, each compound belonging to the three sets of virtual hits was tested at a concentration of 250 µM in a single-point binding assay against fluorescently labelled rhIDO1 (NT650-IDO1) using microscale thermophoresis analysis (MST; Figures S5–S7, Supplementary materials).

Binder compounds were defined as ones inducing a thermophoretic movement of the ligand-bound complex with a fluorescent signal (F-norm) outside the signal value and standard deviations of the buffer vehicle. The fluorescent signal (F-norm) generated from the NLG-919 analogue (**10**), at a concentration of 250 μM, was used as positive control of the binding analysis. One compound of set A was excluded from the analysis due to its tendency to form aggregates, thereby revealing to be a false positive hit compound (VIS539, **15**). As a result, 13 binders were identified in set A, 17 binders in set B, and 5 binders in set C (Table 2; Figures S8–S10, Supplementary materials).

It is worth noting the lack of a direct correlation between the average binding energy scores of the three sets of compounds and the number of binders identified in each of them, suggesting limitations of the scoring function for reliable evaluation of the binding energies of docked ligands to IDO1.

**Figure 6:**
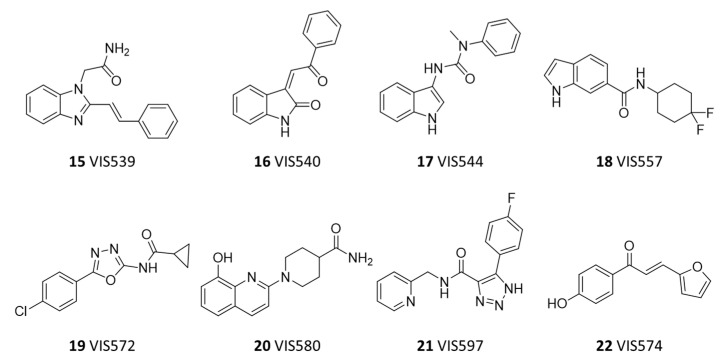
Chemical structures of false positive hit compound (**15**) and confirmed hit compounds (**16**–**22**).

**Figure 7:**
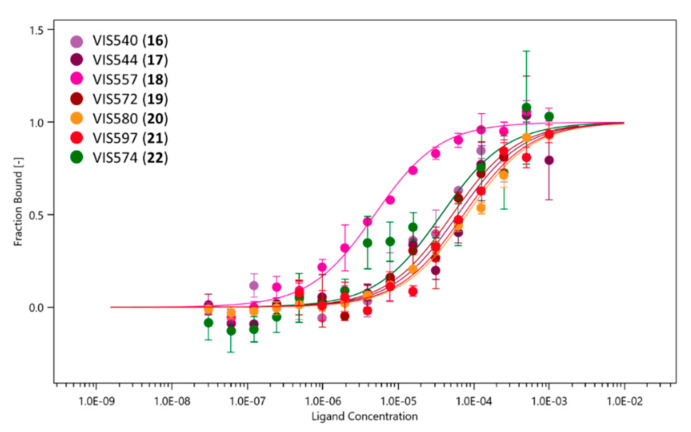
MST binding curves of confirmed hit compounds (**16**–**22**).

Next, compounds classified as binders were tested in full MST assays against NT650-IDO1, using a scalar concentration of each compound. As a result, a dissociation constant (K_d_) lower than 100 µM was determined for seven compounds among the three sets (**16**–**22**, Figure 6 and Figure 7; Table 3), with three of them belonging to set A (compounds **16**–**18**), three to set B (compounds **19**–**21**), and one compound to set C (compound **22**).

Interestingly, IDO1 ligands of set A are endowed with an indole scaffold, including the most potent compound VIS557 (**18**, K_d_ = 4.83 ± 0.61 µM), whereas structures of set B and C ligands include a variety of heterocycles. The inspection of the binding efficiency indices of these ligands allowed for the identification of compounds **16** (BEI = 18), **18** (BEI = 19) and **22** (BEI = 21) as the most promising building blocks for future hit-to-lead optimization studies, with their values resembling the reference IDO1 inhibitor value (**10**, BEI = 20).

Overall, the most productive screening campaigns were obtained with chain A of 6E45 (heme-bound structure) and chain B of 6AZW (inhibitor bound and heme-free structure), whose binding sites are capable of sampling the chemical space located at positive values of PC1 (Figure 5).

### 2.4. Physicochemical Properties

Confirmed IDO1 ligands were profiled for the calculation and experimental determination of their acidic constant (_calc_pKa, _exp_pKa) and n-octanol/water partition coefficient (_calc_logP, _exp_logP). Six compounds showed a _calc_pKa value within the range 2–12 (**16**, **17**, **19**–**22**; Table 4). Four of these compounds (**19**–**22**) were titrated with a potentiometric method to determine the relative _exp_pKa and _exp_logP, using a Sirius T3 instrument. The remaining two compounds (**16**, **17**) were not experimentally analyzed because their _calc_pKa values were too close to the upper detection limit of the Sirius T3 instrument.

As a result, tested compounds span different values of _exp_pKa ranging from 3.9 (**21**) to 8.1 (**19**). Their logP values are range from 1.6 to 2.6, suggesting a good balance between passive permeability and solubility.

A fair correlation can also be observed between the experimental and calculated values, with only compound **21** displaying the _exp_logP value slightly higher than the _calc_logP.

Hence, all ligands were promoted to cellular assays in order to determine their functional activity implying the binding event to IDO1.

### 2.5. Cellular Assay

**Figure 8:**
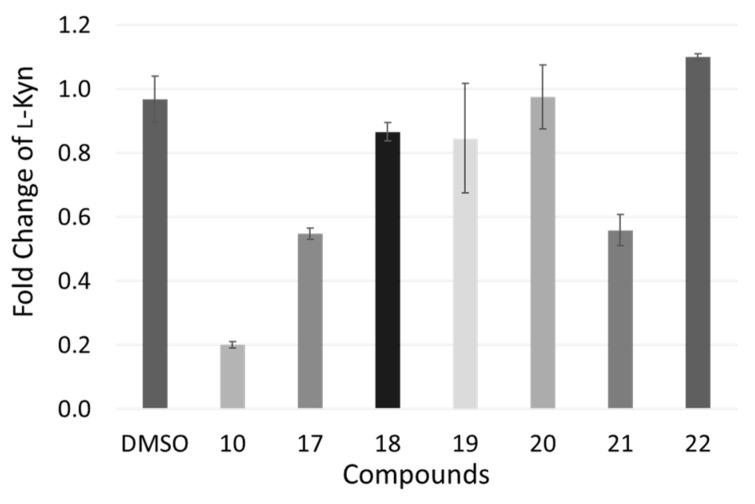
Results of the cellular assay (P1.HTR) of IDO1 inhibition by positive control (**10**) and selected compounds **17**–**22**, expressed as fold change of l-Kyn production.

The functional activity of confirmed IDO1 ligands was estimated in murine mastocytoma cell line (P1.HTR) stably transfected with murine IDO1. Specifically, P1.HTR cells were cultured in presence of each compound at 30 µM concentration. After 16 h, the fold change of l-Kyn concentration was appraised in the culture medium as readout of the inhibition of IDO1 catalytic activity. In agreement with the literature data [45], results show a strong reduction of l-Kyn production after incubation of P1.HTR cells with the reference compound **10** (FC l-Kyn = 0.20 ± 0.01; Figure 8).

Among the seven tested fragments, only compounds **17** (FC l-Kyn = 0.55 ± 0.02) and **21** (FC l-Kyn = 0.56 ± 0.05) showed a significant reduction of l-Kyn production, with another compound being cytotoxic (**16**, data not shown). Accordingly, no correlation could be found between cellular activities and enzymatic binding data of tested compounds. In particular, the two most active compounds in the cellular assay (**17**, **21**) showed poor binding potencies, whereas the most potent IDO1 ligand (**18**) was a weak inhibitor in P1.HTR cells stably expressing IDO1.

Different observations can explain such discrepancies. The inconsistent activity profile of compound **18** may be explained by the presence of an additional binding pocket (*exo* site) in the small domain of IDO1, which is able to bind indole bearing compounds [46,47]. Hence, the binding potency of the indole derivative **18** (K_d_ = 4.83 ± 0.61) against recombinant IDO1 may likely be associated with the interaction with the *exo* site, located outside of the catalytic cleft, affecting less the catalytic activity of IDO1. Conversely, compounds **17** and **21** show significant cellular inhibition activities that are at odds with their poor binding potencies against recombinant IDO1.

In the literature, recent studies have reported that cellular IDO1 may exist in equilibrium between an apo-(heme-free) and holo-(heme-bound) form [35,48,49]. The apo-form can become catalytically competent by adding a heme co-factor and changing into the holo-form. This latter heme-bound form switches from an inactive ferric state to a catalytically active ferrous state of the cofactor group. Therefore, inhibitors may bind to different forms and redox states of cellular IDO1 with variable affinity. This is not the case of recombinant IDO1, wherein only one stable form and redox state of the enzyme exist at the applied experimental conditions, namely the inactive ferric state. Although compounds **17** and **21** show poor binding potencies against the inactive ferric state of recombinant IDO1, this may not be the case against the apo-form and/or the catalytically active ferrous state of cellular IDO1, thereby providing an explanation for their significant activity as enzymatic inhibitors in P1.HTR cells.

Supporting this scenario, the chemical structure of **17** bears a phenylurea moiety that is a key structural feature of some known apo-IDO1 inhibitors (e.g., **14**, BMS-978587), suggesting a preferential interaction of the compound to this enzymatic form. Likewise, compound **21** contains a 1,2,3-triazole moiety. This class of IDO1 inhibitors (e.g., **11**) were recently reported in the literature as able to form a tight binding complex with ferrous IDO1, showing a dissociation constant to the catalytically active ferrous state ten times lower than to the inactive ferric state of IDO1 [37]. Consequently, a disagreement was also found between the inhibition activities of the 1,2,3-triazole compound **11** in the biochemical assay and HEK293 cells overexpressing IDO1, with the cytosolic environment of this cellular system providing an optimal redox condition enabling a higher formation rate of the preferential ferrous target state of IDO1.

## 3. Discussion

Results from our work suggest that the utilization of three distinct conformations of IDO1 in a structure-based screening campaign of a chemical library has augmented the identification and diversity of virtual hits, as indicated by a PCA study with topological descriptors (Figure 5). Thus, diverse size, hydrophobic and hydrogen bonding features of input structures of the binding cleft promote a wider chemical diversity of top scored compounds in molecular docking, thereby representing a tip for IDO1 structure-based screening campaigns.

Upon single-point binding experiments, a higher hit rate (number of binders = 17/23) was found for the holo-IDO1 structure (6E45), featuring a small polar binding site enriched with hydrogen bond donor groups, followed by the apo-IDO1 structure (6AZW), bearing a larger and hydrophobic cleft due to the lack of the heme cofactor (number of binders = 13/23). The holo-structure with the narrowest binding site (6E46) yields the lowest hit rate (number of binders = 5/23). Of note, such a trend of hit rates does not correlate with the average energetic scores of virtual hits to the three structures (Table 2), suggesting that the scoring function of molecular docking is still a pitfall for the correct estimation of the binding energy of compounds to IDO1.

The rank of hit rates is also confirmed after full binding experiments, wherein the holo-IDO1 (6E45) and the apo-IDO1 (6AZW) yield an overall hit rate of 13%, which is three times higher than the holo-IDO1 structure with the narrowest binding site (6E46). Hence, as a further tip, IDO1 structures with a large binding site and hydrophobic character (6AZW), or with a small cleft to which a ligand might be expected to donate hydrogen bonds (6E45), seem to favor the identification of a higher number of IDO1 ligands out of a library of fragment molecules.

Conversely, the discrepancy between biophysical binding data and cellular inhibition activities of confirmed hit compounds is another pitfall that we have found in our screening campaign. In agreement with data from other literature [35,37,48,49], this can be ascribed to the preferential binding of ligands to specific forms and/or redox states of the enzyme in a cytosolic environment which does not occur in biophysical and/or biochemical assay conditions, wherein only one stable form of IDO1 generally exists.

A further explanation may rely on the presence of multiple binding sites in the structure of IDO1 (e.g., *exo* site) that, while detected in the biophysical assay as being involved in ligand interactions, may be silent in a cellular assay if they do not affect the readout parameter, namely the formation of l-Kyn.

## 4. Materials and Methods

### 4.1. Structure Selection and Binding Site Properties

Three IDO1 structures were selected as representative of diverse conformations of the catalytic cleft: 6AZW (chain B) [35], 6E45, (chain A) [36], and 6E46 (chain A) [36]. The atomic coordinates of these structures were retrieved from RCSB PDB (www.rcsb.org; access date 25 July 2020). These structures cover different regions of the molecular recognition space of IDO1, have not been used in previous structure-based screening campaigns, and show the best resolution factor among neighboring structures in the molecular recognition space. The collected structures were processed employing the Protein Preparation Wizard (PPW) tool, as implemented in Maestro (Maestro v12.4, Schrödinger, LLC, New York, NY, USA). In particular, hydrogen atoms were added, and the internal geometries of the protein were optimized with a coordinate displacement restrain on heavy atoms set to 0.3 Å. Atoms of the heme group and protein were parametrized using the OPLS3e force field. This force field has been reported to show improved performance in predicting protein−ligand binding affinities [50]. Accordingly, the choice of the OPLS3e force field was made expecting a better estimate of protein−ligand binding affinities and favoring a quicker process of parametrization for the cofactor and protein atoms.

SiteMap (SiteMap v12.5, Schrödinger, LLC, New York, NY, USA) was next employed to calculate binding site properties, which include volume (Å^3^), balance in hydrophobic/hydrophilic character of the site (hydrophobic balance), and the degree to which a bound ligand is expected to make hydrogen-bond donor interactions rather than hydrogen-bond acceptor interactions (Don./Acc.). The binding pocket was defined on the center of mass of the inhibitor for 6AZW and 6E46, and the center of mass of the substrate for 6E45. Binding site descriptors were calculated with default settings of SiteMap, using a grid size of 0.7 Å and a minimum number of 15 site points.

### 4.2. Virtual Screening Protocol

A library of 44,000 fragments was downloaded from Life Chemicals Inc. (lifechemicals.com, access date 12 November 2018), annotating the molecular weight (*M*_W_) and polar surface area (PSA) that were calculated using Canvas (Schrödinger Release 2020-2: Canvas, Schrödinger, LLC, New York, NY, USA).

To carry out docking studies, all compounds were prepared using LigPrep (LigPrep v8.7, Schrödinger, LLC, New York, NY, USA), generating all ionization and tautomeric states at pH = 7 ± 2.

A virtual screening campaign was performed for each of the three IDO1 structures (chain A of 6E45, chain B of 6AZW, and chain A 6E46), using Glide docking (Glide v8.4, Schrödinger, LLC, New York, NY, USA) [51,52], with the standard precision (SP) scoring function and the expanded sampling mode.

Considering the fragment size of the compounds, a docking protocol previously validated for fragments was employed [53]. The grid box was defined considering the binding poses of the co-crystallized ligands. In 6AZW and 6E46, the grid was built on the center of mass of the co-crystallized inhibitors. In 6E45, the grid was centered using the atomic coordinates of the co-crystallized substrate. The inner box was sized 10 × 10 × 10 Å. Docked compounds showing a Gscore lower than −4 kcal/mol were selected from each of the three virtual screening campaigns and submitted to three separate clustering analyses. Such an arbitrarily cutoff value of Gscore allowed retrieval of the following rates of top scored compounds amid successfully docked ligands for each IDO1 structure: 99.4% (6AZW), 76.3% (6E45), and 68.3% (6E46). Histograms showing the distribution of Gscore values for the successfully docked ligands in each IDO1 structure are shown in Figure S4 of the Supplementary material.

Clustering analysis was performed by calculating the radial Morgan ECFPs and applying the K-means method with 10 runs composed of 30 steps each. A total of 23 clusters were obtained for each virtual screening campaign. A representative compound was selected in each cluster according to the criteria of better binding energy (Gscore, kcal/mol) and purchase availability, yielding a total of 69 compounds for biophysical testing. The diversity covered in the “chemical space” by the 69 compounds was analyzed by performing a principal component analysis (PCA) with n.199 topological descriptors calculated for each molecule. The PCA was performed using Canvas (Canvas v4.4, Schrödinger, LLC, New York, NY, USA).

### 4.3. Biophysical Binding Assays

Microscale thermophoresis (MST) assay was instrumental to confirm the binding properties of the selected 69 compounds against recombinant human IDO1 (rhIDO1) protein. At this aim, rhIDO1 was purchased from Proteros Biostructures GmbH (PR-0113) and labelled using the Protein Labelling Kit RED-NHS (NT-650, NanoTemper Technologies GmbH, München, Germany), according to the previously reported protocol [45]. The real concentration of the protein (4 µM) and the degree of labelling (DoL) were determined by absorption spectroscopy [54]. The MST signal for each capillary was recorded using 20% LED Power, choosing the Manual 19/20s hot region. The pool of selected compounds was screened using a single-point MST assay, and each experiment was repeated three times employing Premium Coated capillaries. Particularly, compounds were tested at a fixed concentration of 250 μM against fluorescently labelled rhIDO1 at a final concentration of 50 nM. The reaction mixture was diluted in MST buffer (50 mM Tris-HCl, 150 mM NaCl, 10 mM MgCl_2_, 0.05% Tween-20) supplied by NanoTemper Technologies, adding 2% DMSO and 2 mM dithiothreitol (DTT). All experiments were performed in the presence of a positive control (**10**, NLG919 analogue; Selleckchem catalogue n.S7111) and a vehicle (2% DMSO). The NLG919 analogue (**10**) is an IDO1 inhibitor with an IC_50_ of 38 nM in cell-based assay [26], and a dissociation constant (K_d_) of 3.3 µM in MST assay [45]. As a rule of thumb, molecules were classified as binders when they produced a F-norm signal value higher than the vehicle standard deviation [55].

Full-point MST assays were carried out on binders identified in the single-point assay, with the aim of determining their K_d_ values against rhIDO1. Each compound was tested starting with the 1:1 dilution from its maximum reachable concentration in MST buffer with 4% DMSO. The experiment was performed adding 10 µL of 100 nM labelled rhIDO1 to each PCR tube obtaining a final concentration of 50 nM in MST buffer with 2 mM DTT and 2% DMSO. K_d_ values were extrapolated by compound concentration-dependent changes of red rhIDO1 F-norm signals. The Binding Efficiency Index (BEI) was then calculated using the following the Equation (1) [56]:

BEI = pK_d_/*M*_W_ (kDa) (1)

### 4.4. Physicochemical Property Evaluations

Acidic constants (pKa) and octanol/water partition coefficients (logP) of confirmed hit compounds were firstly calculated using the pKa and logP plugins of the program Marvin (Marvin v20.11, ChemAxon, Budapest, Hungary). Then, the experimental appraisal of compounds with calculated pKa values (_calc_pKa) within the range of pKa 2–12 was performed using the Sirius T3 potentiometric method (Pion Inc. Ltd., Forest Row, East Sussex, UK) [57].

In order to avoid kinetic solubility issues during the dissolution stage in ionic strength adjusted (ISA) water (KCl 0.15 M), the selected compounds were dissolved in 100% DMSO with a concentration of 50 mM and sampled as an organic solution. The experiments were set up with Sirius T3 control software (Sirius T3 v1.1.3.0, Pion Inc. Ltd., Forest Row, East Sussex, UK), specifying the volume of the stock solution to be used, molecular weight, number of expected pKa and their calculated values (_calc_pKa), predicted logP (_calc_logP), and titration mode (from pH = 2 to 12 for basic compounds, and from pH = 12 to 2 for acidic compounds). To avoid issues of poor solubility during the titration in ISA water, the pKa analyses were carried out in triplicate with different ratios of organic cosolvent methanol and water (50%, 40%, and 30% methanol). Considering the modification of water’s dielectric constant in the presence of an organic solvent, the pKa at 0% of co-solvent was obtained through the method of Yasuda–Shedlovsky (YS) extrapolation to zero [58]. The validity of the analysis was assessed with the r^2^ of the YS method, and results were considered acceptable with an r^2^ ≥ 0.9. Titration results were analyzed with Sirius T3 refine software (Sirius T3 v1.1.3.0, Pion Inc. Ltd., Forest Row, East Sussex, UK). Octanol/water partition coefficient (logP) was measured through a potentiometric titration in a biphasic system, using ISA water and n-octanol. Specifically, Sirius T3 control software uses three evaluation models of experimental logP, whose choice is based on the calculated logP values (_calc_logP < 1, “Low-logP” method; 1 < _calc_logP < 3, “Medium-logP” method; _calc_logP > 3, “High-logP” method). Accordingly, the “Medium-logP” method was selected to evaluate the experimental logP (_exp_logP) of the selected compounds. The titration was performed in triplicate and the raw data were elaborated using Sirius T3 refine software. The quality of the analysis was evaluated by comparing the titration curve of each analysis by calculating a root mean square deviation (RMSD). All the analysis yielded RMSD values < 0.5.

### 4.5. Cellular Assay

A cellular assay was performed to assess the functional activity of confirmed hit compounds against IDO1. Briefly, P1.HTR cells, a highly transfectable clonal variant of mouse mastocytoma P815 [59], were cultured in Iscove’s modified Dulbecco’s medium supplemented with 10% fetal calf serum (FCS), 1 mM glutamine and penicillin/streptomycin at 37 °C in a humidified 7% CO_2_ incubator. P1.HTR cells were transfected by electroporation with plasmid constructs coding for murine IDO1 (P1.IDO1). A stable transfectant cell line was obtained by puromycin selection. Cells at the concentration of 0.25 × 10^6^ cell/mL were incubated with 30 µM of compounds for 16 h, using a culture medium containing l-Trp at a concentration of 78.4 µM and a HEPES buffer at pH 7.2.

A cell culture incubated with an equivalent volume of DMSO (the vehicle in which compounds were solubilized) was used as a reference control. After the incubation, supernatants of cell cultures were recovered, and kynurenine concentration was detected by the HPLC method. Every cell assay was conducted in triplicate. Results were expressed as the mean ± standard deviation of the kynurenine fold change (l-Kyn FC), meaning the ratio between kynurenine concentration secreted in the supernatant of the compound-treated versus vehicle-treated cells. The detection of l-Kyn concentrations was performed by using a PerkinElmer, series 200 HPLC instrument. A Kinetex C18 column (250 × 4.6 mm, 5 μm, 100 A; Phenomenex, CA, USA), maintained at the temperature of 25 °C and a pressure of 1800 PSI, was used. A sample volume of 300 μL was injected and eluted by a mobile phase containing 10 mM NaH_2_PO_4_ pH 3.0 (99%) and methanol (1%), with 1 mL/min flow rate. l-Kyn was detected at 360 nm by an UV detector. The software TURBOCHROM 4 was used for evaluating the concentration of l-Kyn in samples by means of a calibration curve. The detection limit of the analysis was 0.05 μM.

## 5. Conclusions

This research study has shown that the identification and diversity of hit compounds in structure-based screening campaigns can be reinforced when implementing more than one protein conformation to simulate the diverse forms that a target protein may assume in cells. Therefore, with the exploitation of different IDO1 chain conformations, diverse ligand compounds were attained. However, a first pitfall was encountered with the estimation of the docking scores and the poor correlation of these energetic values to the dissociation constants (K_d_) of hit compounds.

The hit rate is also affected by other pitfalls represented by the availability of only a single enzymatic form capable of maintaining stability during the biophysical assay, as well as the use of a single readout in the cellular assay. In this regard, future screening campaigns could benefit from the use of a battery of biophysical assays that are optimized with different experimental conditions to stabilize distinct forms of IDO1 in each assay. Moreover, the design of cellular assays with multiple redouts tailored to detect additional downstream signaling functions of IDO1 could prove beneficial to get insights into the putative functions of the catalytically silent sites of the enzyme.

## Figures and Tables

**Table 1:** Properties of binding site for the selected IDO1 structures.

Structure ^1^	Hydrophobic Balance ^2^	Don./Acc. ^3^	Volume (Å^3^) ^4^
6AZW	3.60	1.31	291.21
6E45	1.11	2.97	98.78
6E46	1.52	0.75	85.41

^1^ PDB codes corresponding to the selected entries of IDO1 structures in the Research Collaboratory for Structural Bioinformatics (RCSB) database. ^2^ Ratio between the hydrophobic and hydrophilic character of the binding site. ^3^ Degree to which a ligand is expected to donate (Don./Acc. > 1) or accept (Don./Acc. < 1) hydrogen bonds. ^4^ Size of the binding site expressed as site volume according to SiteMap criteria.

**Table 2:** Average polar surface area (PSA), mode of the number of hydrogen bond donor (HB-Don.) and acceptor (HB-Acc.) groups, and average binding energy scores (GScore) of the three set of selected compounds. The number of binders from the microscale thermophoresis (MST) single-point assay is also indicated.

Virtual Hits	PSA Avg. ± St.Dev (Å^2^)	HB-Don. Mode	HB-Acc. Mode	GScore Avg. ± St.Dev (kcal/mol)	N. Binders
Set A (6AZW)	60.95 ± 14.02	1	2	−9.36 ± 0.31	13
Set B (6E45)	78.88 ± 20.01	2	3	−6.09 ± 0.70	17
Set C (6E46)	75.52 ± 17.19	1	2	−7.89 ± 0.84	5

**Table 3:** Dissociation constants (K_d_) ± confidence values and binding efficiency index (BEI) of the selected IDO1 ligands.

Compounds	K_d_ (μÌM)	Set	*M*_W_	BEI ^1^
**10**	2.05 ± 0.17	-	282	20
VIS540 (**16**)	37.51 ± 11.52	A	249	18
VIS544 (**17**)	59.25 ± 22.89	A	265	16
VIS557 (**18**)	4.83 ± 0.61	A	278	19
VIS572 (**19**)	51.94 ± 18.35	B	263	16
VIS580 (**20**)	80.69 ± 10.60	B	271	15
VIS597 (**21**)	73.26 ± 16.25	B	297	14
VIS574 (**22**)	36.98 ± 16.80	C	214	21

^1^ Binding efficiency index defined as BEI = pK_d_/*M*_W_ (see methods).

**Table 4:** Experimental and calculated physicochemical properties of selected IDO1 ligands.

Compounds	_exp_logP	_calc_logP	_exp_pKa	_calc_pKa
**10**	3.2	3.0	6.7	6.3
VIS540 (**16**)	n.d. *	2.8	n.d. *	11.2
VIS544 (**17**)	n.d. *	3.1	n.d. *	11.5
VIS557 (**18**)	n.d. *	2.4	n.d. *	-
VIS572 (**19**)	2.1	2.3	8.1	7.2
VIS580 (**20**)	1.9	1.8	5.7	5.9/9.3
VIS597 (**21**)	2.0	1.6	3.9/6.3	4.1/9.2
VIS574 (**22**)	2.6	2.6	7.8	7.9

* n.d. = not determined (out of the range of values of Sirius T3, pH 2–12).

## Data Availability

Not applicable.

## Supplementary Materials

The following supporting information can be downloaded at: https://www.mdpi.com/article/10.3390/ijms23073981/s1.
