# Supplementary material for: Critical Assessment of a Structure-Based Screening Campaign for IDO1 Inhibitors: Tips and Pitfalls
Source: Int J Mol Sci. 2022 Apr 2;23(7):3981. doi: 10.3390/ijms23073981 (PMC8999677; doi:10.3390/ijms23073981)
Supplement: Supplementary file 1 [file ijms-23-03981-s001.zip › ijms-1653968-supplementary.pdf]

Table of Contents:

Figure S1. Binding site properties of the catalytic cleft in the IDO1 structure 6AZW. Solvent accessible surfaces are generated with SiteMap and color-coded according to total area (grey surface), hydrogen bond acceptor area (red surface), hydrogen bond donor area (violet surface), hydrophobic area (yellow surface).

Figure S2. Binding site properties of the catalytic cleft in the IDO1 structure 6E45. Solvent accessible surfaces are generated with SiteMap and color-coded according to total area (grey surface), hydrogen bond acceptor area (red surface), hydrogen bond donor area (violet surface), hydrophobic area (yellow surface).

Figure S3. Binding site properties of the catalytic cleft in the IDO1 structure 6E46. Solvent accessible surfaces are generated with SiteMap and color-coded according to total area (grey surface), hydrogen bond acceptor area (red surface), hydrogen bond donor area (violet surface), hydrophobic area (yellow surface).

Figure S4. Histograms showing the distribution of Gscore values for the successfully docked ligands in each IDO1 structure (**a**, 6AZW; **b**, 6E45; **b**, 6E46).

Figure S5. Single point assay of MST binding experiments of virtual hit compounds (set A) from IDO1 structure 6AZW. The blue filled circle shows the positive control (concentration of 250 µM), the green filled circle shows negative control (buffer vehicle).

Figure S6. Single point assay of MST binding experiments of virtual hit compounds (set B) from IDO1 structure 6E45. The blue filled circle shows the positive control (concentration of 250 µM), the green filled circle shows negative control (buffer vehicle).

Figure S7. Single point assay of MST binding experiments of virtual hit compounds (set C) from IDO1 structure 6E46. The blue filled circle shows the positive control (concentration of 250 μM), the green filled circle shows negative control (buffer vehicle).

Figure S8. Chemical structures of n.13 binders from IDO1 structure 6AZW.

Figure S9. Chemical structures of n.17 binders from IDO1 structure 6E45.

Figure S10. Chemical structures of n.5 binders from IDO1 structure 6E46.

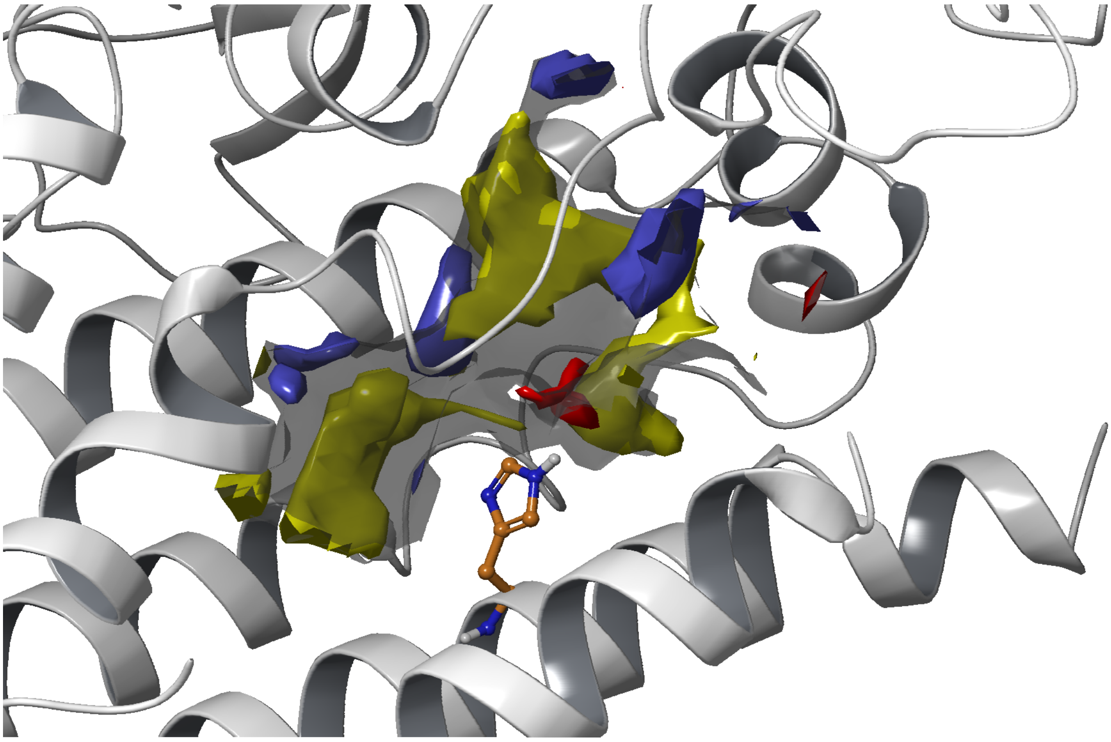

Figure S1. Binding site properties of the catalytic cleft in the IDO1 structure 6AZW. Solvent accessible surfaces are generated with SiteMap and color-coded according to total area (grey surface), hydrogen bond acceptor area (red surface), hydrogen bond donor area (violet surface), hydrophobic area (yellow surface).

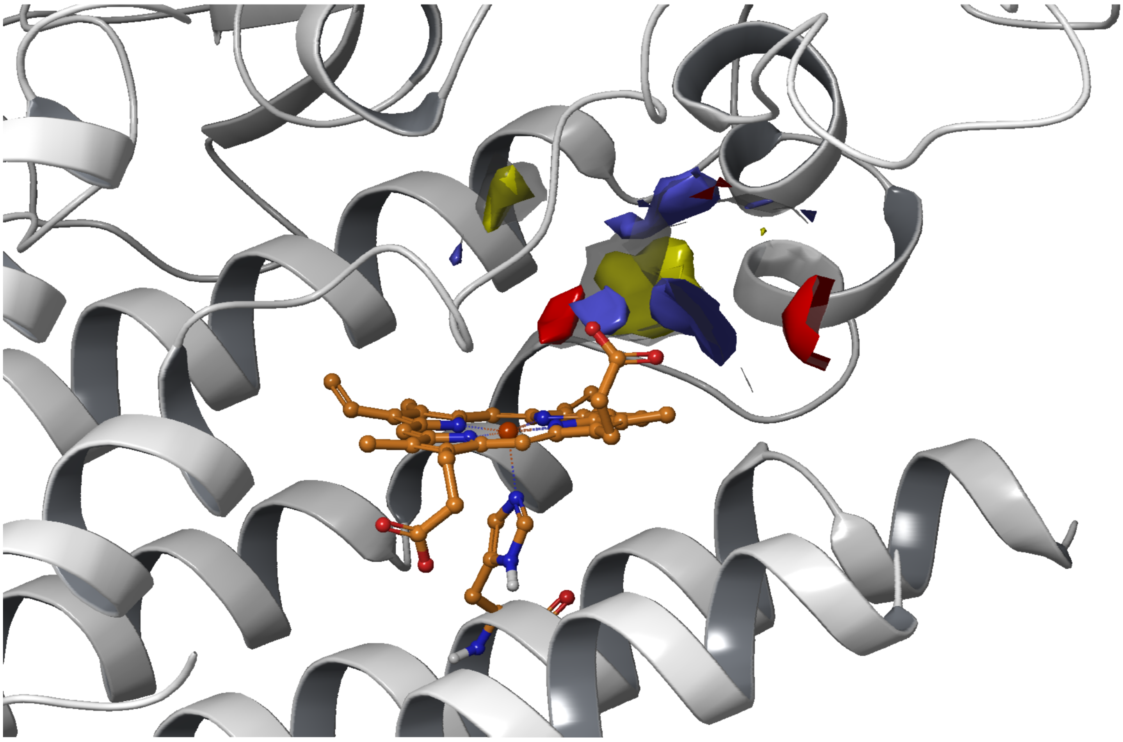

Figure S2. Binding site properties of the catalytic cleft in the IDO1 structure 6E45. Solvent accessible surfaces are generated with SiteMap and color-coded according to total area (grey surface), hydrogen bond acceptor area (red surface), hydrogen bond donor area (violet surface), hydrophobic area (yellow surface).

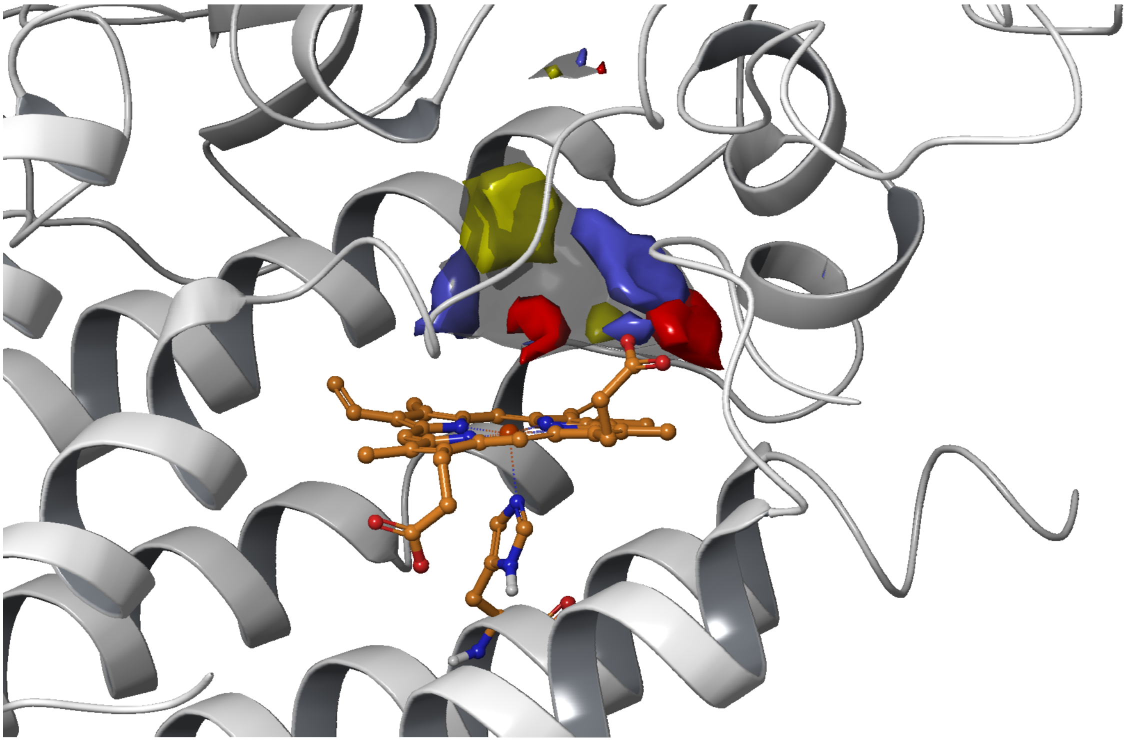

Figure S3. Binding site properties of the catalytic cleft in the IDO1 structure 6E46. Solvent accessible surfaces are generated with SiteMap and color-coded according to total area (grey surface), hydrogen bond acceptor area (red surface), hydrogen bond donor area (violet surface), hydrophobic area (yellow surface).

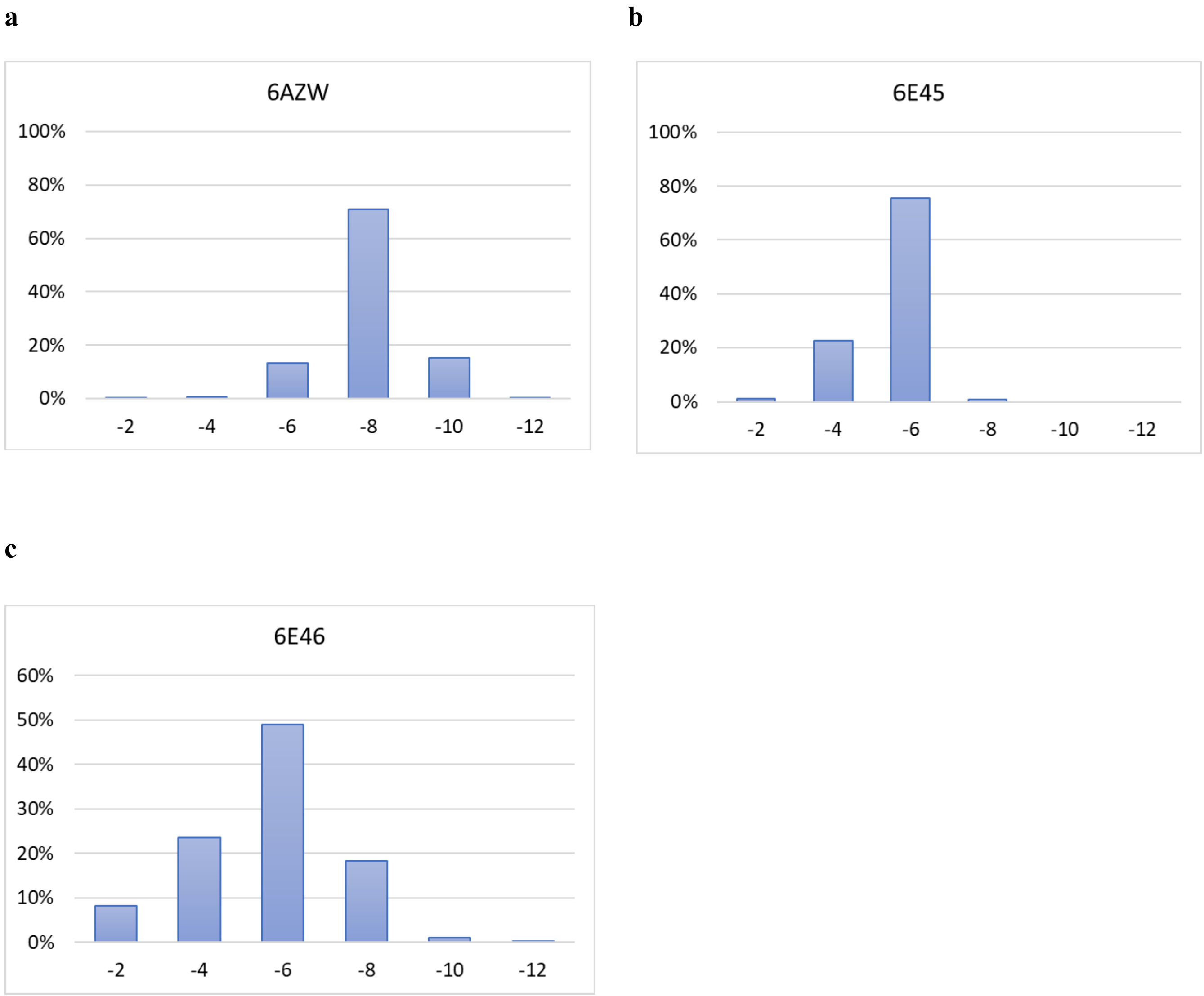

Figure S4. Histograms showing the distribution of Gscore values for the successfully docked ligands in each IDO1 structure (**a**, 6AZW; **b**, 6E45; **b**, 6E46).

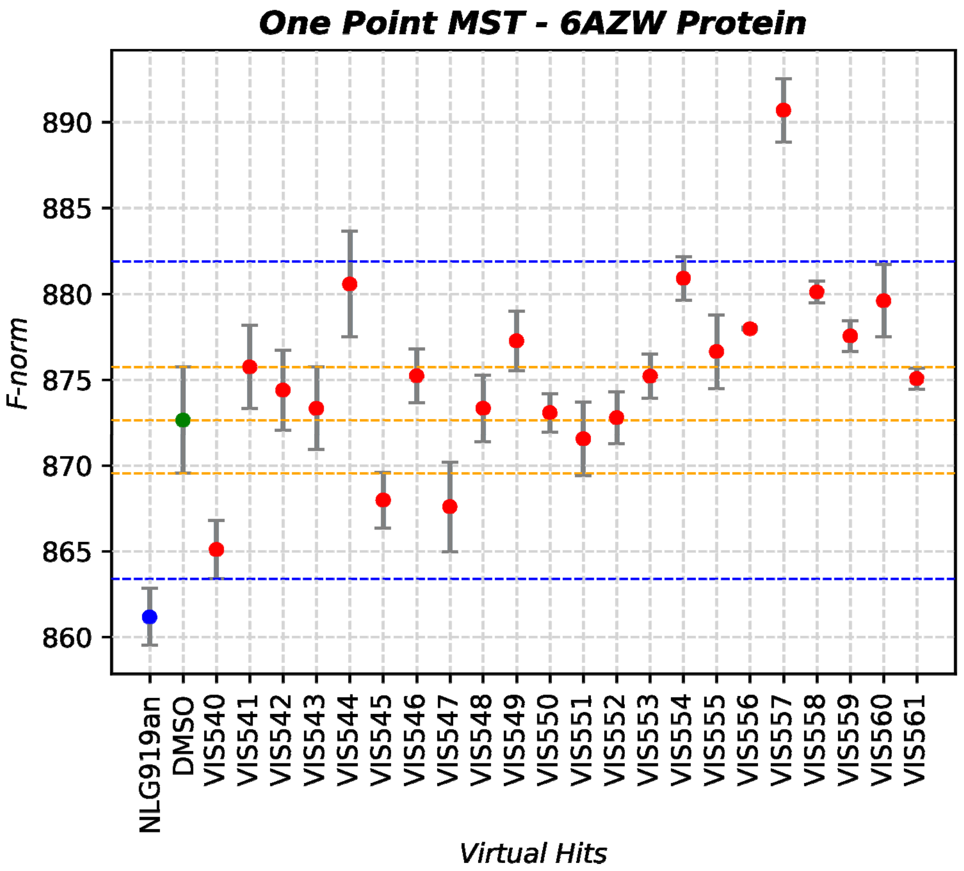

Figure S5. Single point assay of MST binding experiments of virtual hit compounds (set A) from IDO1 structure 6AZW. The blue filled circle shows the positive control (concentration of 250 µM), the green filled circle shows negative control (buffer vehicle).

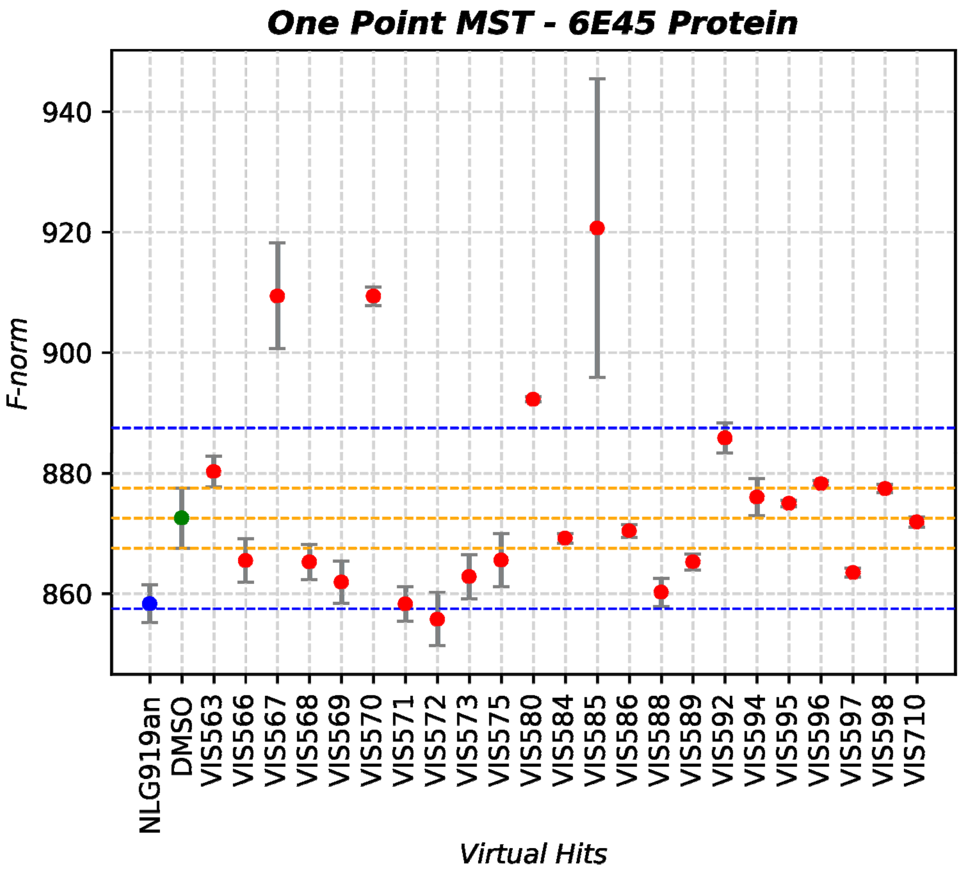

Figure S6. Single point assay of MST binding experiments of virtual hit compounds (set B) from IDO1 structure 6E45. The blue filled circle shows the positive control (concentration of 250 µM), the green filled circle shows negative control (buffer vehicle).

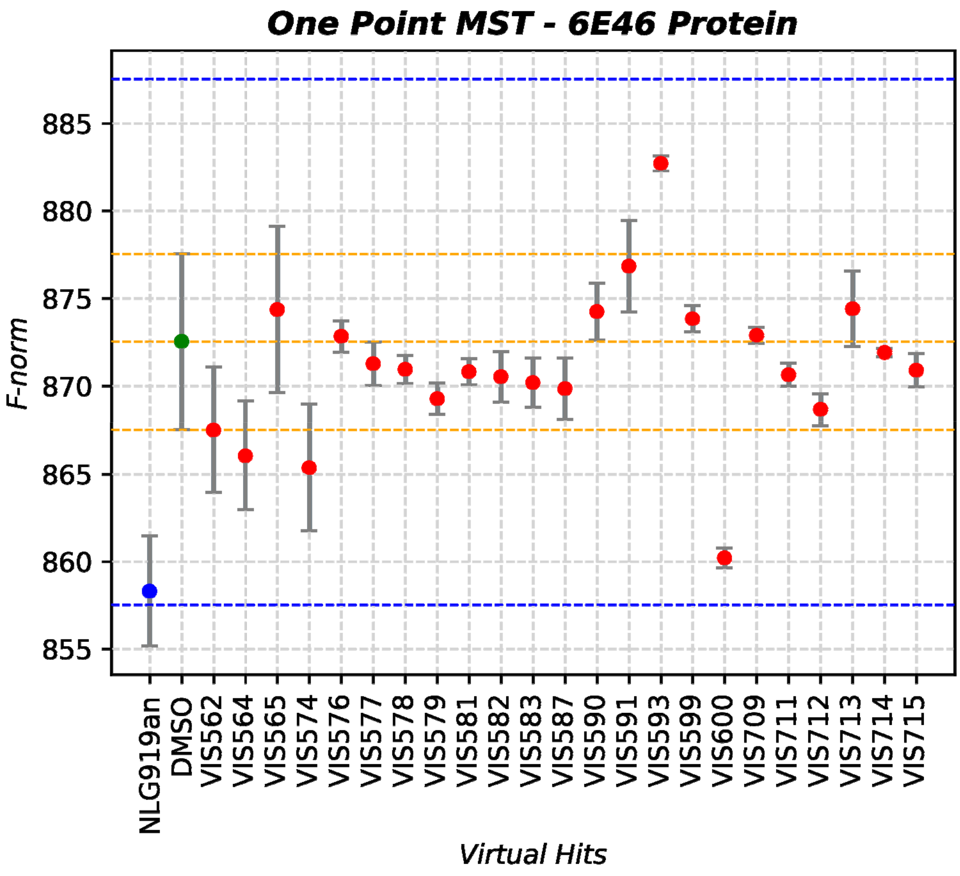

Figure S7. Single point assay of MST binding experiments of virtual hit compounds (set C) from IDO1 structure 6E46. The blue filled circle shows the positive control (concentration of 250 µM), the green filled circle shows negative control (buffer vehicle).

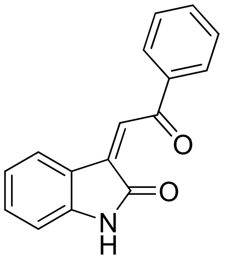

VIS540 (**16**)

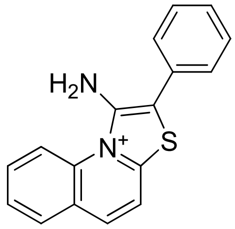

VIS541

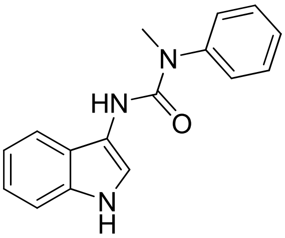

VIS544 (**17**)

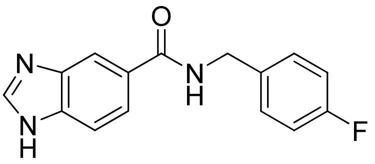

VIS545

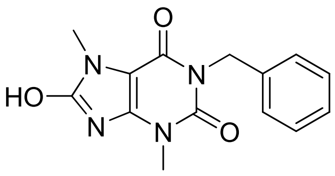

VIS546

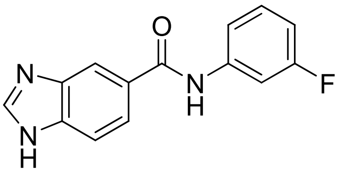

VIS547

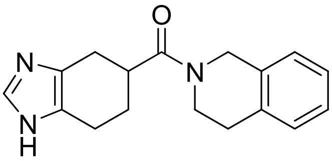

VIS549

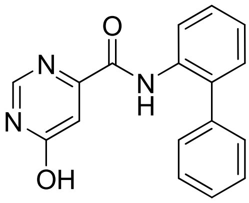

VIS554

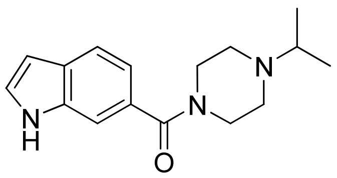

VIS555

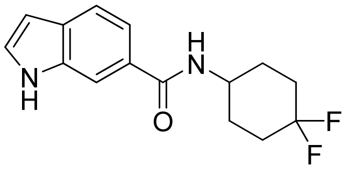

VIS557 (**18**)

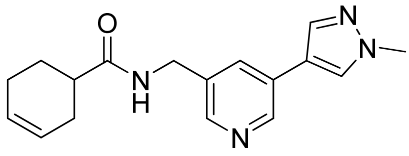

VIS558

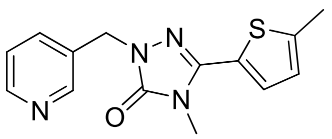

VIS559

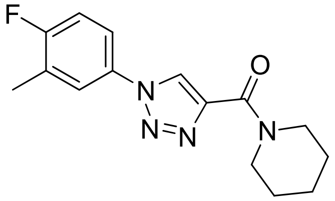

VIS560

Figure S8. Chemical structures of n.13 binders from IDO1 structure 6AZW.

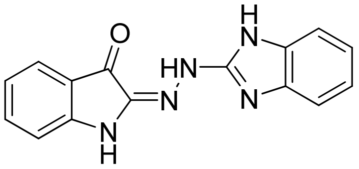

VIS563

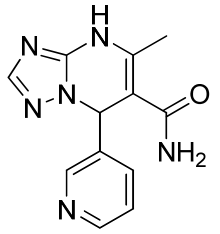

VIS566

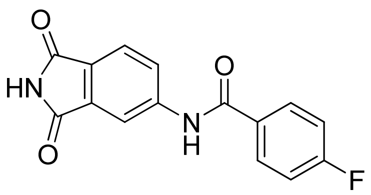

VIS567

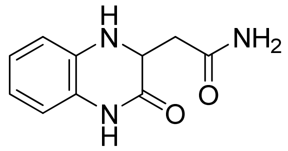

VIS568

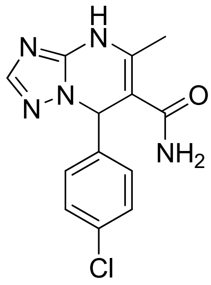

VIS569

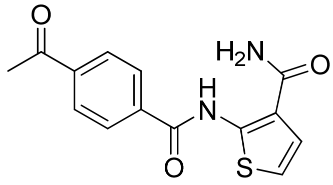

VIS570

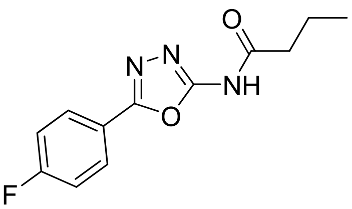

VIS571

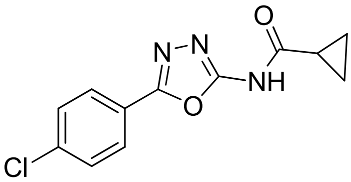

VIS572 (**19**)

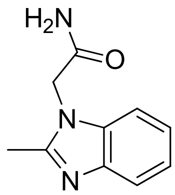

VIS573

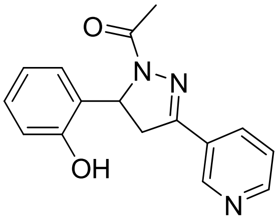

VIS575

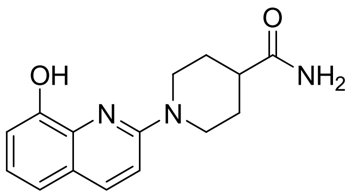

VIS580 (**20**)

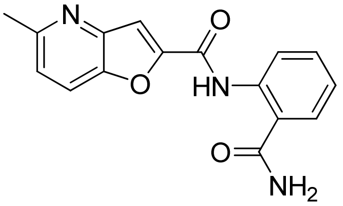

VIS585

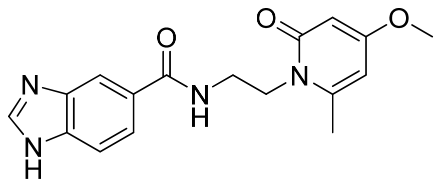

VIS588

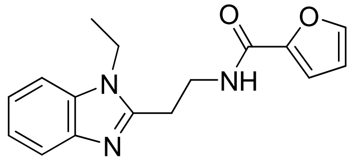

VIS589

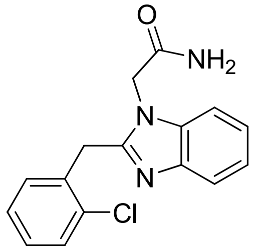

VIS592

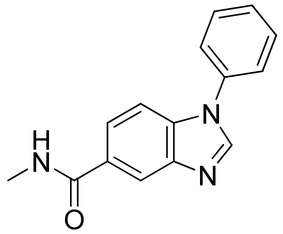

VIS596

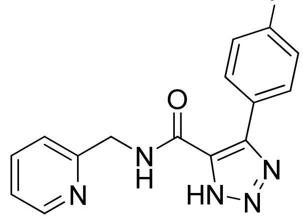

VIS597 (**21**)

Figure S9. Chemical structures of n.17 binders from IDO1 structure 6E45.

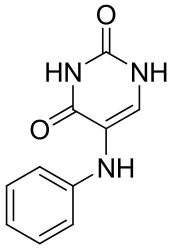

VIS562

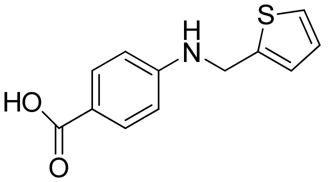

VIS564

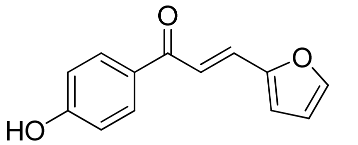

VIS574 (**22**)

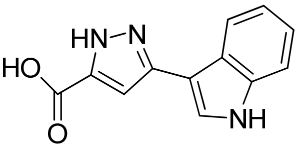

VIS593

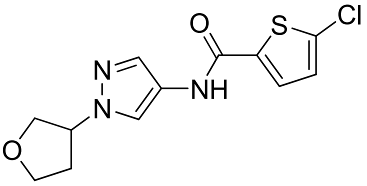

VIS600

Figure S10. Chemical structures of n.5 binders from IDO1 structure 6E46.
